# Tackling Ischemic Reperfusion Injury With the Aid of Stem Cells and Tissue Engineering

**DOI:** 10.3389/fphys.2021.705256

**Published:** 2021-09-17

**Authors:** Mauricio Zamorano, Rodrigo L. Castillo, Jorge F. Beltran, Lisandra Herrera, Joaquín A. Farias, Christian Antileo, Cristobal Aguilar-Gallardo, Adalberto Pessoa, Yolanda Calle, Jorge G. Farias

**Affiliations:** ^1^Department of Chemical Engineering, Universidad de La Frontera, Temuco, Chile; ^2^Department of Internal Medicine, Universidad de Chile, Santiago, Chile; ^3^Facultad de Ingeniería y Ciencias, Universidad Adolfo Ibíñtez, Santiago, Chile; ^4^Hematological Transplant and Cell Therapy Unit, Hospital Universitario y Politécnico La Fe, Valencia, Spain; ^5^Department of Biochemical and Pharmaceutical Technology, School of Pharmaceutical Sciences, University of São Paulo, São Paulo, Brazil; ^6^Department of Life Sciences, Whitelands College, University of Roehampton, London, United Kingdom

**Keywords:** ischemia reperfusion injury, stem cells, tissue engineering, 3D culture, IRI mechanism

## Abstract

Ischemia is a severe condition in which blood supply, including oxygen (O), to organs and tissues is interrupted and reduced. This is usually due to a clog or blockage in the arteries that feed the affected organ. Reinstatement of blood flow is essential to salvage ischemic tissues, restoring O, and nutrient supply. However, reperfusion itself may lead to major adverse consequences. Ischemia-reperfusion injury is often prompted by the local and systemic inflammatory reaction, as well as oxidative stress, and contributes to organ and tissue damage. In addition, the duration and consecutive ischemia-reperfusion cycles are related to the severity of the damage and could lead to chronic wounds. Clinical pathophysiological conditions associated with reperfusion events, including stroke, myocardial infarction, wounds, lung, renal, liver, and intestinal damage or failure, are concomitant in due process with a disability, morbidity, and mortality. Consequently, preventive or palliative therapies for this injury are in demand. Tissue engineering offers a promising toolset to tackle ischemia-reperfusion injuries. It devises tissue-mimetics by using the following: (1) the unique therapeutic features of stem cells, i.e., self-renewal, differentiability, anti-inflammatory, and immunosuppressants effects; (2) growth factors to drive cell growth, and development; (3) functional biomaterials, to provide defined microarchitecture for cell-cell interactions; (4) bioprocess design tools to emulate the macroscopic environment that interacts with tissues. This strategy allows the production of cell therapeutics capable of addressing ischemia-reperfusion injury (IRI). In addition, it allows the development of physiological-tissue-mimetics to study this condition or to assess the effect of drugs. Thus, it provides a sound platform for a better understanding of the reperfusion condition. This review article presents a synopsis and discusses tissue engineering applications available to treat various types of ischemia-reperfusions, ultimately aiming to highlight possible therapies and to bring closer the gap between preclinical and clinical settings.

## Ischemia

Ischemia is a severe condition that is characterized by interruption and reduction in blood supply, including oxygen (O), to organs and tissues. This is usually due to a clog or blockage in the arteries that feed the affected organ; it is an intrinsic component of severe peripheral artery disease and organ transplantation. Ischemia induces an imbalance between the metabolic supply and demand of tissues, producing local hypoxia, accumulation of cellular by-products, and acidification of the cellular environment (Eltzschig and Eckle, 2011; Slegtenhorst et al., 2014; Dua and Lee, 2016). In due process, adenosine triphosphate (ATP) falls to critical levels. Thus, membrane pumps fail which leads to intracellular sodium and calcium build-up. In addition, mitochondrial complexes are compromised where there is a rise in the production of reactive oxygen species (ROS) above its physiological levels. Each tissue can withstand different periods of ischemia prior to displaying an appreciable dysfunction, or evidencing injury. Brain, heart, kidney, and intestine are among the most susceptible tissues with 20–30 min being sufficient, while skeletal tissue displays a critical ischemic period of some hours (Kalogeris et al., 2012). However, once the critical period is surpassed (depending on cell type and organ), cell swelling, membrane damage, and/or cell death follow (Kalogeris et al., 2012; Slegtenhorst et al., 2014). Consequently, minimizing hypoperfusion duration is essential to salvage the affected tissues done through the restoration of blood flow, including O, and nutrient supply. Current therapies rely on prompt restoration of blood flow through percutaneous coronary intervention or the use of fibrinolytic agents, among others (Wu et al., 2018). However, reperfusion itself does not come disencumbered.

## Ischemia-Reperfusion Injury

Ischemia-reperfusion injury is often prompted by the profound local and systemic inflammatory reaction, as well as oxidative stress, that accompanies reoxygenation and enhances the final tissue-damage (Eltzschig and Eckle, 2011). Pathophysiological events associated with reperfusion affect a wide array of organs and conditions, including heart failure, stroke, myocardial stunned/hibernating/infarction, wounds, lung, renal, liver, and intestinal damage or failure, which are concomitant, in due process, with disability, morbidity, and mortality. In addition, ischemia-reperfusion injury (IRI) damage is not restricted to the specific ischemic tissue, a remote organ injury could follow reperfusion, a systemic inflammatory response syndrome, as well as multiple organ dysfunction (Kalogeris et al., 2012). It has been established that the damage produced by longer periods of ischemia is aggravated by reoxygenation, while an adaptative response is provided by short courses of preconditioning ischemia. Through this preconditioning therapy, activation of intrinsic cell-survival programs makes tissues resistant to the detrimental effects of reperfusion (Kalogeris et al., 2012). Other therapeutic strategies for IRI management include antioxidant therapy, anticomplement therapy, antileukocyte therapy, or the administration of nitric oxide (NO), which may also be from NO donors or drugs that boost NO release (Collard and Gelman, 2001). The full mechanism encompassing IRI remains not fully defined despite the impressive discoveries done through more than 30 years of research invested in understanding this condition (Kalogeris et al., 2012). Therefore, novel approaches for this purpose and pioneer therapies to address this condition are in need (Wu et al., 2018). Here, tissue engineering (TE) is proposed as an adequate platform to study, tackle IRI, and propose the next generation of IRI treatments.

## Mechanisms of Ischemia and Ischemia-Reperfusion Injury

**Figure 1:**
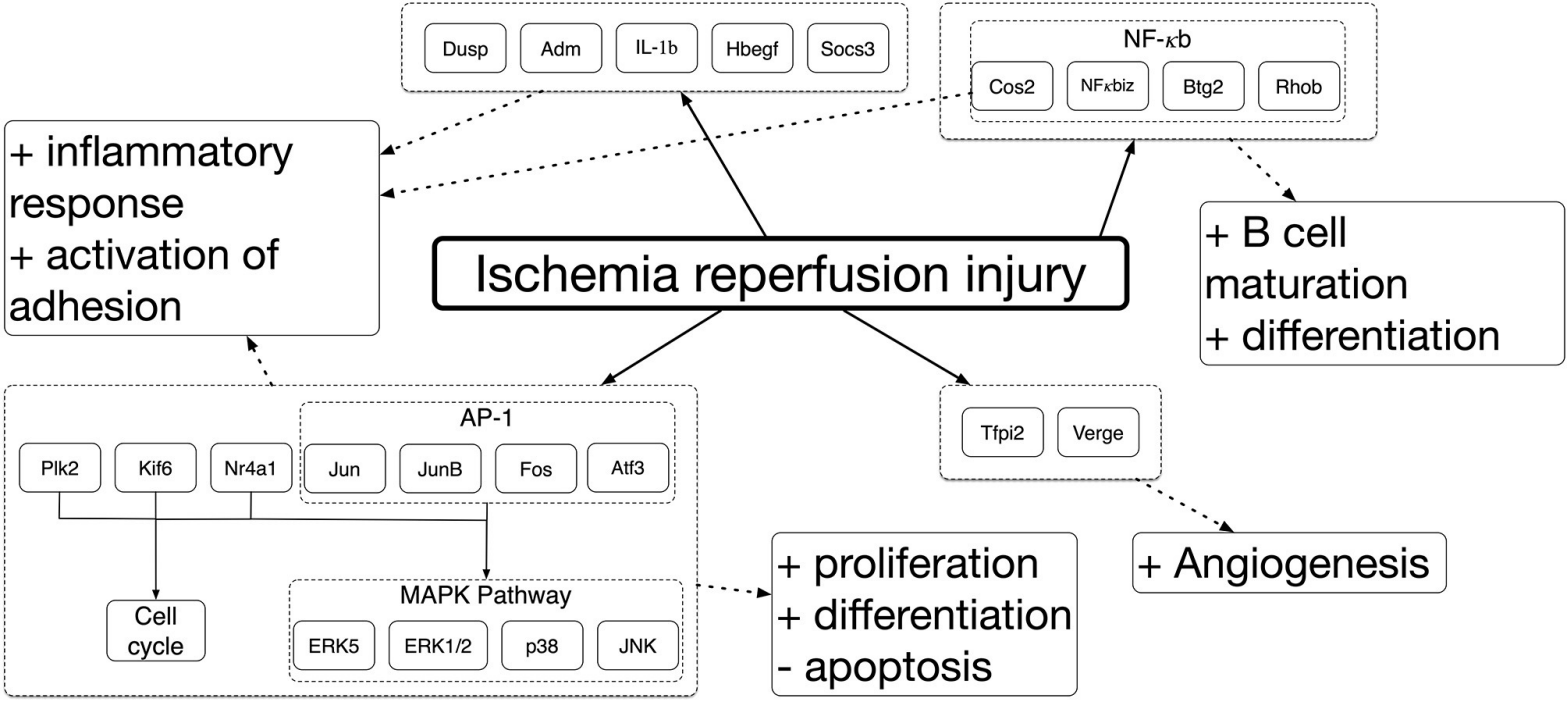
Schematic depiction of the IRI mechanism, and MAPK pathway modulation.

Ischemia and IRI differentially induce the expression of a large number of genes that drive the response across different tissues. Genome-wide gene expression profiling has been used to establish ischemic and IRI mechanisms. Deoxyribonucleic acid microarrays were used to study the genes involved in ischemic damage and repair different organs in animal models. It was seen that genes expressed were varied among organs. Despite these mechanisms being complex and organ-specific (Kalogeris et al., 2012), around 1,000 sequences were differentially expressed in most cases when comparing to controls. Between these, more than 70% were over-expressed, and the rest were repressed (Paoni et al., 2002; Chang et al., 2015). Among the over-expressed genes, pathways related to the inflammatory reaction and hypoxia, such as cell proliferation, were identified. Correspondingly, a decreased expression of transcripts encoding proteins involved in mitochondrial activity, adhesion, and contraction were identified. Similar studies have been done to analyse gene expression across different organs during IRI. The results displayed several commonly upregulated genes amongst kidney, intestine, and skeletal muscle, associated with identified mitogen-activated protein kinase (MAPK), nuclear factor kappa-light-chain-enhancer of activated B cells (NFκB), and extracellular signal-regulated kinase 1/2 (ERK1/2) pathways (Chang et al., 2015; Zheng et al., 2017; Shao et al., 2018). These results are summarized in Figure 1 [modified from Chang et al. (2015)]. A brief summary of the mechanisms driving ischemia and IRI will be presented. However, the readership should refer to several excellent reviews for a more in-depth description of these mechanisms (Collard and Gelman, 2001; Eltzschig and Eckle, 2011; Kalogeris et al., 2012; Wu et al., 2018; Soares et al., 2019).

Adaptation to the hypoxic environment accompanying ischemia induces dysfunction of the electron transport chain in mitochondria, which trigger a cascade of events that cause severe cellular malfunction and tissue damage. The decrease in mitochondrial ATP prompts anaerobic metabolism, sodium-potassium/calcium (Na-K/Ca) pumps malfunction, and ribosomal detachment. The metabolic changes are followed by decreased ATP production, cellular antioxidants availability, an increase in lactic acid, and acidification. Due to Na-K/Ca pumps dysfunction, Na and K exchange is hindered. The former being accumulated inside the cells and the latter being kept outside. Sodium-exchanger/calcium pumps failure ensues, while hydrogen (H), Na, and Ca ions accumulate in the cytoplasm. The accumulated ions cause hyperosmolarity, cell swelling, and further acidification (Bompotis et al., 2016). Then an impairment in enzyme activity and nuclear chromatin clumping is induced. In addition, the detachment of ribosomes reduces protein synthesis (Kalogeris et al., 2012; Forrester et al., 2018). These events translate into the reduced activity of nicotinamide adenine dinucleotide phosphate (NADPH) oxidase (NOX) enzymes, adenosine monophosphate (AMP)-activated protein kinase (AMPK), and B-cell lymphoma 2 (Bcl2) proteins. Furthermore, these events translate to the activation of numerous genes regulators of O homeostasis within cells, such as hypoxia-inducible factor (HIF), vascular endothelial growth factor (VEGF), and MAPK, e.g., ERKs, c-Jun N-terminal kinases (JNKs), and the p38 MAPKs (Jiménez-Castro et al., 2019). In due process, these alter angiogenesis, vascular remodeling, cell migration, and produce high ROS yield, ROS-mediated autophagy, and inflammation (Ziello et al., 2007; Mokhtari and Lewis, 2014; Forrester et al., 2018; Koeppen et al., 2018). In addition, the distribution of protein kinase C (PKC) changes, modifying cell proliferation, and apoptosis (Mokhtari and Lewis, 2014).

The onset of reoxygenation produces a transition from a hypoxic to a normoxic environment, which is linked in due process to a burst of ROS, and oxidative stress. This negative effect is exerted by oxidants, such as superoxide anion radicals, or hydrogen peroxide (H_2_O_2_). Reactive oxygen species can bind directly to key regulatory proteins or participate in the production of reactive nitrogen oxide species (RNOS), such as peroxynitrite. This then leads to the distribution of cell signaling and redox circuits. Sources of cellular superoxide production are xanthine oxidoreductase (XOR) system, NADPH oxidase system, cytochrome P450 oxidases uncoupled nitric oxide synthase (NOS) system, hemoglobin, and myoglobin. These will be implicated depending on the tissues involved and the severity of the injury (Wu et al., 2018).

The shift to anaerobic glycolysis and reduced mitochondrial respiration caused by ischemia, along with the resurfacing of oxygen in the environment, will further increase ROS production, inflammatory reaction, produce nitrogen reactive species (NRS), and RNOS (Rodrigo et al., 2013; Forrester et al., 2018; Barzegar et al., 2019). Nitrogen reactive species, such as NO, and ONOO^−^, are formed through the oxidation of arginine, catalyzed by NOS; the reduction of nitrite, or nitrate, through the action of XOR; or the action of mitochondrial cytochrome C oxidase under hypoxia. The low concentrations of NRS produced by endothelial NOS fulfill physiological functions and interact with other species to form nitrosyl complexes with proteins that transport iron and heme. These complexes prevent the formation of ferryl-heme radicals by H_2_O_2_. The higher concentrations of NRS produced by inducible NOS are detrimental, resulting in the production of RNOS, and harmful to macromolecules, i.e., membrane lipids, proteins, and DNA. These NRS affect cellular signal transduction, producing pro-inflammatory mediators, activate apoptosis, necrosis, and autophagy pathways. Ultimately, expanding the ischemic damage, as well as decreasing the availability of protective NO (Kalogeris et al., 2012; Chen et al., 2013).

In an attempt to re-establish the normal cytosolic pH after ischemic acidification, Ca regulation will be altered. Cells will exchange H ions for Na. These Na ions will subsequently be exchanged for Ca. Reoxygenation will further increase the cytosolic Ca overload, producing detrimental alterations in Ca homeostasis, proton gradients, mitochondrial failure, hypercontracture, and proteolysis, which will contribute to the overall ROS production (Bompotis et al., 2016). In addition, tumor necrosis factor (TNF)-like cytokines, ROS, and Ca activate receptor-interacting protein (RIP) kinases; together with NFκB, they will drive a cascade of events that will induce the expression of various pro-inflammatory genes, produce an immune response, and activate necroptosis (Kalogeris et al., 2012; Liu et al., 2017).

## Mimicking Ischemia-Reperfusion Injury in 3D *ex vivo* Settings

**Figure 2:**
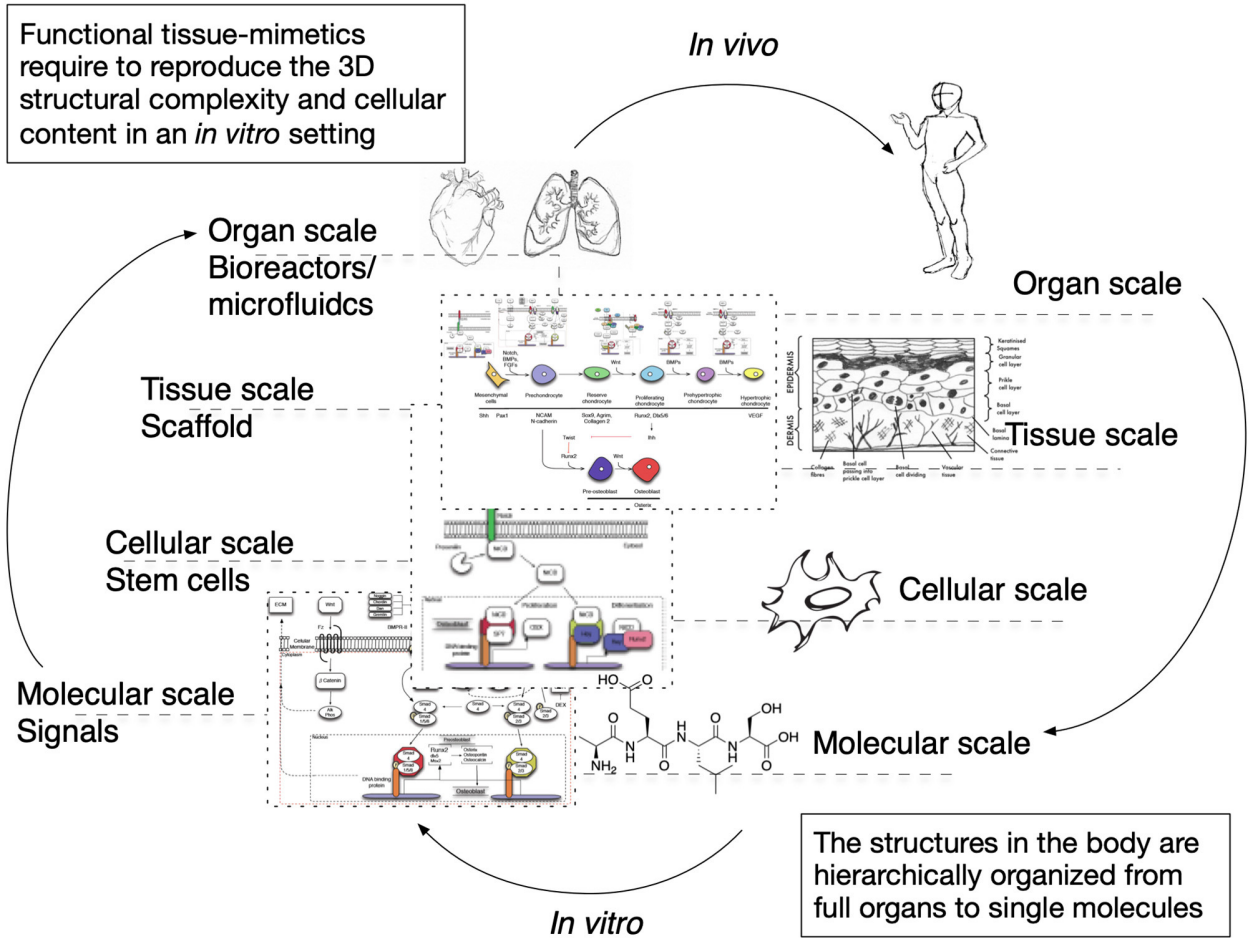
Hierarchical organization of organs, and the building-up of TE-tissue-mimetics.

**Figure 3:**
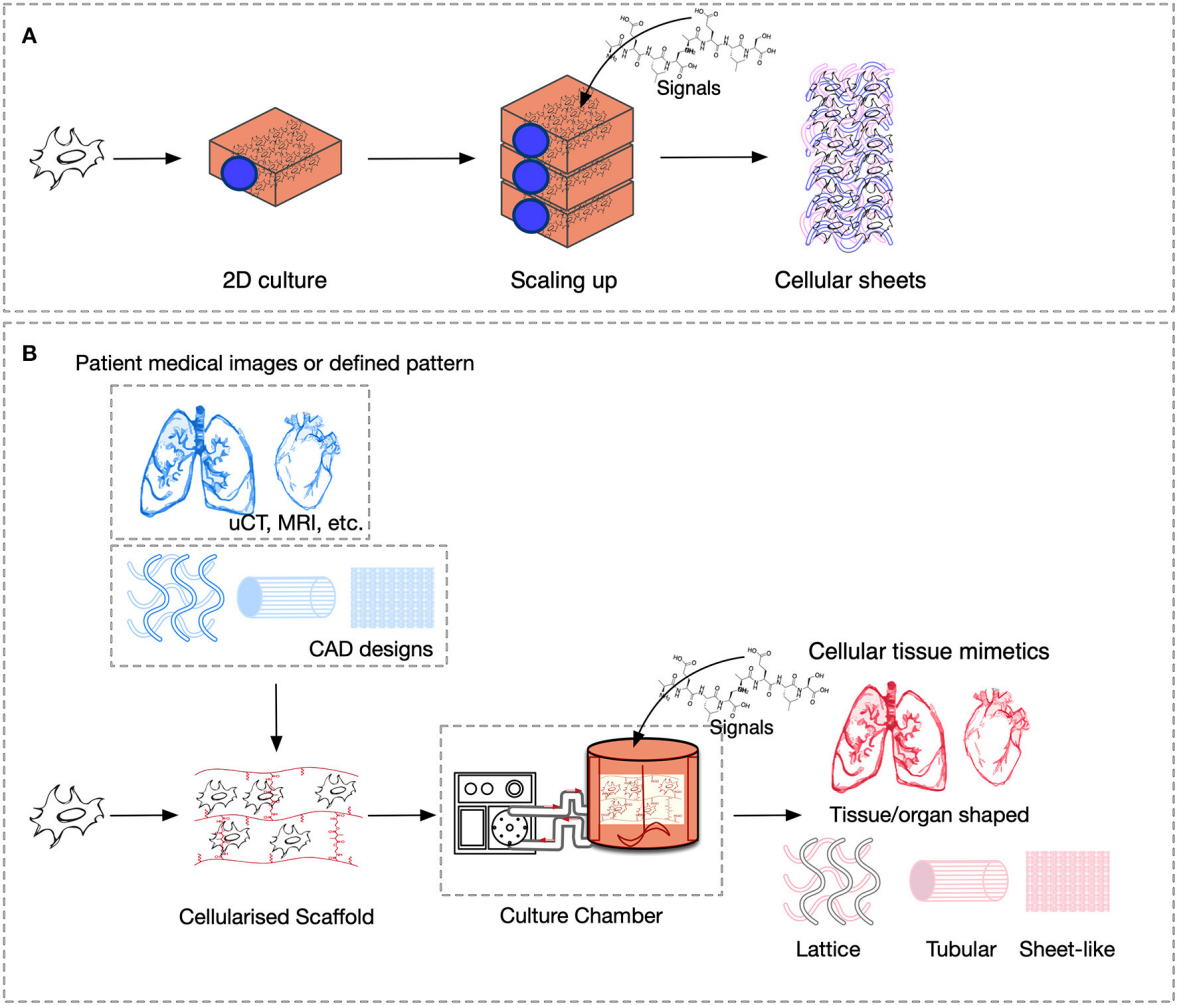
Comparison of culture platforms, where scheme **(A)** represents 2D culture, and **(B)** 3D culture.

The cues that drive IRI, the complex crosstalk between all molecular pathways involved, and its tissue-specific features variability must be fully understood and fine-tuned in order to provide robust applications and therapeutics. Its intricate nature has been tried to mimic in two-dimensional *in vitro* (2D), and animal-based *in vivo* models. In general, medical research heavily relies on these models when testing new therapies and/or therapeutics. This is also true for IRI research wherein 2D static monolayered culture systems have allowed the building of the foundations for the understanding of disease cues. In these systems, cells are grown attached to a supportive plane in their basal surface and are exposed to culture media in their upper face. Consequently, cells in the basal area receive limited contact with nutrients and signals, developing heterogeneously. Cells allocated into planar cell culture flasks become flattered, divide abnormally, and lose their differentiated phenotype (Baker and Chen, 2012). Thus, many findings in 2D are not reproducible *in vivo* or tissue explants. In addition, researching cells alone, cannot provide a comprehensive, and a suitable platform to mimic disease. In tissues, cells are enclosed in a three-dimensional (3D) complex microarchitecture, coordinated by intricate signaling dynamics. This 3D environment is subjected to mechanical stresses and in constant interaction with adjunct tissues. To minimize the gap between the 2D platform, and the *in vivo* domain, animal models have been put to use and have proven to be an irreplaceable source of data for understanding IRI. Albeit these models represent a critical platform and the gold standard for disease studies, which are costly, time-consuming, and often not reproducible in human studies (Meigs et al., 2018). Additionally, animal testing is associated with ethical concerns; all ethical committees will require researchers to **replace** these studies with other methods, when possible. They are suggested to **reduce** the number of animal subjects to the lowest and **refine** the studies to minimize the impact on said subjects. Thus, finding alternative methods to achieve such a benchmark is on-demand (the 3**R**s principles; Festing and Wilkinson, 2007). The efforts to achieve tissue-mimicry, decrease animal testing, the development of 3D static, and dynamic culture systems have derived into the TE principia. This approach employs stem cells (SCs), 3D scaffolding types of machinery, biological cues, and bioprocess tools, such as biophysical cues, in an attempt to better-mimic the host local and global tissue interactions. In addition to this improved environment for cell expansion and maturation, nutrients exchange, waste removal between cells, and culture media could be incorporated through culture media perfusion. See Figure 2 for schematics of the hierarchical organization of organs and the building-up of TE-tissue-mimetics. It has already been demonstrated by several studies that this improved culture environment allows cells to maintain most of their characteristics and markers under prolonged culture periods. This is due to a closer mimicry of tissue features, in comparison with 2D. These features include cellular heterogeneity and niche, extracellular matrix (ECM) deposition, cell-cell signaling, cellular interactions, growth kinetics, and gene expression (Godara et al., 2008; Sailon et al., 2009; Baker and Chen, 2012; Costa et al., 2016). This strategy, if provided by tissue-specific features and adequate cells, then it would deliver experimentally accessible human testing models to study the biological processes of IRI while limiting the ethical considerations that animal models elucidate. See Figure 3 for a comparison between 2D and 3D platforms.

## Stem Cells With Application in Ischemia-Reperfusion Injury Research

Using living cells to produce functional tissue substitutes will enable to model, replace, repair, and/or regenerate both healthy and failing tissues; in application to IRI research and treatment, there is a necessity in growing, fabrication, implantation of tissues *ex vivo*, and/or the growth of these *in vivo* (Langer and Vacanti, 1993). From a cellular perspective, the players involved in IRI models should be cells, extracellular matrix (ECM), signaling, cellular dynamics, and the interactions between these.

**Figure 4:**
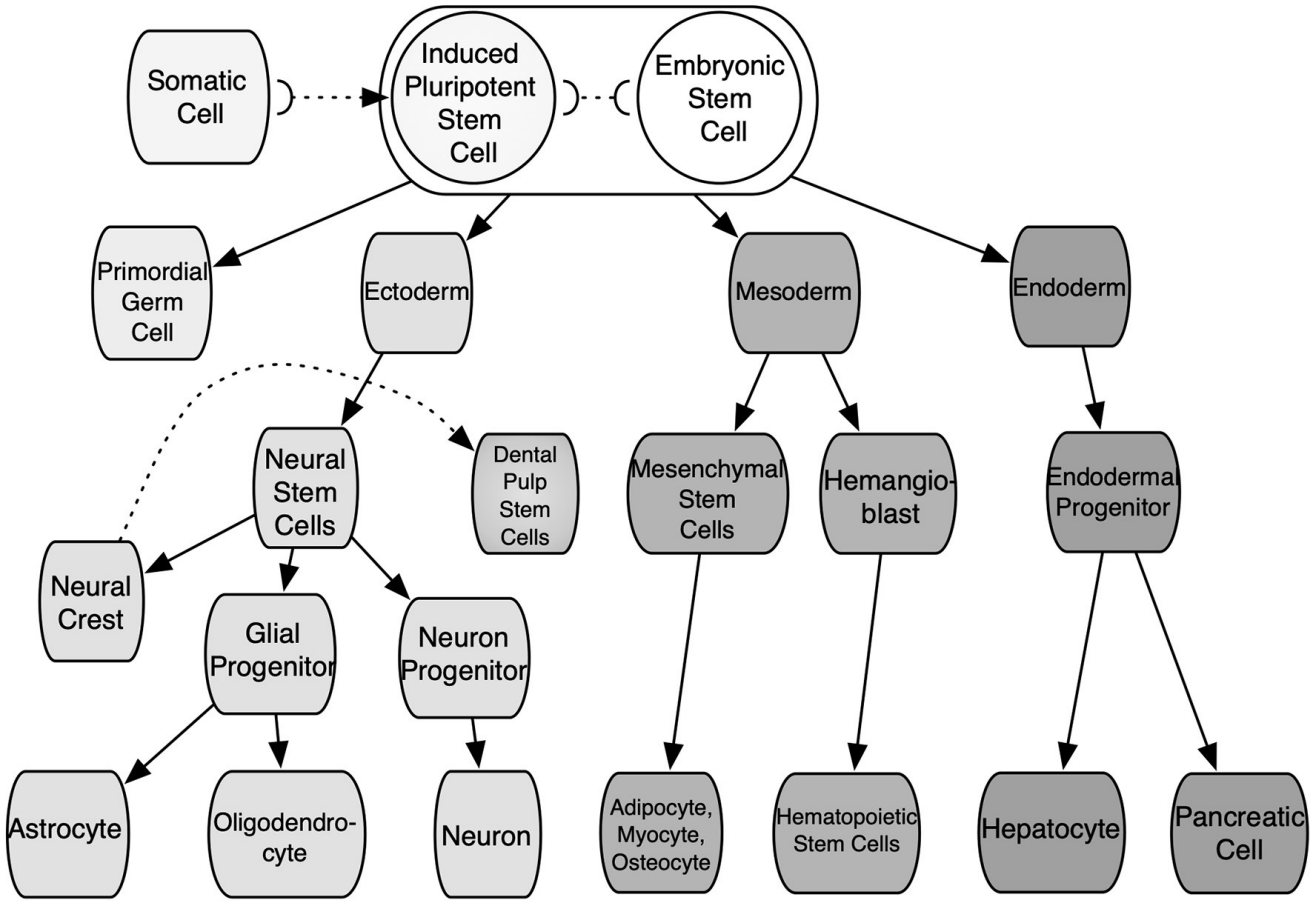
Stem cell sources.

The straightforward alternative of cells with the potential for researching IRI would be autologously sourced from patients and tissue-specific. These cells would not present an immunogenic reaction from the body and could be further expanded *in vitro* to increase their numbers. These would not require differentiation and would be expected to be fully functional. Yet these cells are associated with various shortcomings: time-consuming production; invasive and damaging extraction for the patient and/or donor; extraction yields low cell numbers; poor expansion. The scaling-up of these cells is usually shadowed by senescence and phenotype loss. Moreover, tissue diaschisis associated with IRI would provide cells with heterogeneous functionality and quality. Xenogeneic cells could solve some of the aforementioned hindrances. However, these are related to immunogenic reactions and phenotypic mismatch. Thus, the adequate source of cells should (1) not cause any immune response from the body toward them (or their by-products), (2) be able to proliferate and yield high cell numbers without losing quality, and (3) have ease of access and extraction. In addition, these should have the capability to produce specific functional cell types through differentiation. Stem cells such as embryonic stem cells (ESCs), induced pluripotent stem cells (iPSCs), adult stem cells, i.e., mesenchymal stem cells (MSCs), share some of these features and are best suited than any kind of somatic cells to produce 3D *ex vivo* IRI mimics. These cells can be obtained from patient donors or cell banks. Nonetheless, the differentiation and expansion potential differs among them. These features depending largely on the developmental stage and source of cells, as some of them are constrained to specific cell lines or a closely related family of cells. These are classified according to their differentiation potency. First, totipotent stem cells are able to generate all the cell lineages of a creature, including embryonic, somatic, and germ cells; in mammalians, only the zygote and the first cleavage blastomeres are part of this group. Pluripotent stem cells (PSCs) are capable of indefinite expansion and can give rise to the three germ layers, such as endoderm, mesoderm, ectoderm, and any cell lineage further in the developmental stage. Found in this group are ESCs and iPSCs. The former are derived from the inner cell mass of blastocyst-stage embryos, as well as embryonic germ cells from the developing fetal gonadal ridge. While the latter are manufactured by reprogramming *in vitro* adult somatic cells to an embryonic-like state by the introduction, and forced expression of the main genes and factors of pluripotency (Heng et al., 2004; Vallier et al., 2009; Walia et al., 2012). Thus, iPSCs can be obtained from modified cells from patients or cell banks. These features make pluripotent cells some of the most useful cells for TE. Nonetheless, ESCs are associated with teratoma formation, their differentiation is very difficult to control, which elicits ethical considerations due to the destruction of the embryos in their extraction process (Placzek et al., 2009). On the other hand, iPSCs eliminate ethical constraints but harbor capabilities underneath ESCs. Second, adult multipotent stem cells are a heterogeneous group of cells capable of giving rise to multiple cell types within a specific lineage. In addition, these possess a limited self-renewal capability. These are undifferentiated cells found throughout the adult body in specific niches. Mesenchymal stem cells have been isolated from the connective tissue of almost every organ, such as bone marrow-derived mesenchymal stem cells (BM-MSCs), umbilical cord blood-derived mesenchymal stem cells (UCB-MSCs), adipose tissue-derived stem cells (ADSCs), muscle-derived stem cells (MDSCs), and dental pulp stem cells (DPSCs), among others (Polak et al., 2008; Placzek et al., 2009). All these imply a role as a storage and regenerative pool for the various mesenchymal tissues at the postnatal stage. These present immunomodulatory effects, both *in vitro* and *in vivo* (Fibbe et al., 2007). See Figure 4 for a depiction of stem cell sources. Donor parameters such as age, life habits, gender, and medical record also affect MSCs quality. Third, unipotent stem cells are able to give rise to one specific cell type, and present the lowest self-renewal capability, among SCs, i.e., spermatogonial stem cells. In general, the lineage-specific differentiation of SCs can be directed under specific culture conditions by manipulating the microenvironment where they reside and with biological cues (Walia et al., 2012). These parameters can be managed by bioprocessing design tools. However, since each source of SCs differ in expansion and differentiation rate, factors secretion, and stimulation mechanisms, there is not one defined path to follow and the selected cell source and the specific tissue to mimic will ultimately determine the selected culture strategy and the culture media. Thus, different 3D platforms coupled with SCs, such as spheroids, organoids, hydrogels, scaffolds, and microfluidic systems (either suspended or embedded in a matrix culture), have been pioneered to attempt to mimic aspects of healthy and sick tissues. Ultimately, these 3D platforms allow to design novel SCs-based therapies and therapeutics in due process.

## Spheroids and IRI

Spheroids are micro-size cell aggregates that make use of the cell-to-cell adhesion features of SCs. These features allow cells to form 3D constructs which lack any type of anchorage medium (non-scaffold-based) and are mainly comprised of ECM. Thus, this platform can replicate some of the main characteristics of tissue microarchitecture. Spheroids can be formed by different techniques such as liquid overlay, hanging-drop, rotating wall vessels/spinner flasks generated, microfluidic-based assembly, and magnetic levitation (Ryu et al., 2019). Applications based on spheroids can be customized by selecting the adequate cells, fine-tuning size, culture media composition, and by modulating biophysical cues. This technique can be automated to enable, scalability, reproducibility, and minimize manual handling (Bartosh et al., 2010). In addition, by modulating spheroid size, oxygen availability (and thus, normoxia/hypoxia) in the spheroid core can be modulated to a certain extent. Such platforms have been used to study the mechanism of IRI, the role of SCs in the regeneration process, and to produce biologics with potential therapies in renal (Xu et al., 2016; Zhang, X. et al., 2021), hepatic (Sun et al., 2018; Olander et al., 2019), intestinal (Gonzalez et al., 2019), cardiovascular (Chen et al., 2007; Suzuki et al., 2019; Li et al., 2020; Sfriso and Rieben, 2020), lung (Pagano et al., 2017), skin (Park et al., 2017), fat (Mineda et al., 2015), and neural (Davenport Jones et al., 1998; Serganova et al., 2004; Fu et al., 2015; Liu et al., 2015; Chen et al., 2019) reperfusion damage.

A myocardial IRI kidney model, utilizing porcine coronary artery endothelial cells (PCAECs) and spheroids *in vitro* and *in vivo in* mice, was established to recapitulate the IRI-ROS mechanism (Chen et al., 2007). The model allowed us to explore the protein kinase B (Akt), ERK1/2 activation mechanism, as well as the onset of angiogenesis/VEGF expression (before and after reoxygenation). In addition, it allowed the studying of the role of NADPH oxidase as a modulator and potential therapeutic for IRI treatment.

An acute kidney injury (AKI) model, utilizing raw human ADSCs from liposuction aspirates, and spheroids *in vitro*, and *in vivo* in rats, was established to assess their effect in reperfusion injury (Xu et al., 2016). *In vitro* results highlighted that spheroid culture produced higher levels of ECM proteins, such as collagen I, fibronectin, and laminin, as well as better anti-apoptotic and anti-oxidative capacities, compared with 2D. Additionally, the paracrine secretion of cytokines, such as angiogenic factors VEGF and FGF2, anti-apoptotic factors endothelial growth factor (EGF) and HGF, anti-oxidative factor IGF, and the anti-inflammatory protein TNF-alpha induced protein 6, was higher than 2D. *In vivo* results followed a similar trend and displayed increased survival and paracrine effects than 2D. Notably, when spheroids were transplanted into IRI-AKI rat models, these proved to perform better against apoptosis, reducing tissue damage, promoting vascularisation, and improving renal function compared with 2D.

To study intestinal IRI, porcine intestinal epithelial stem cells (ISCs) were harvested after a prolonged duration of vascular occlusion to guarantee deep epithelial loss. Afterward, spheroid cultures were established to determine the impact of different durations IRI (1–4 h) on ISCs *in vitro* and in the ischemic crypt and the contribution of these cells to mucosal repair (Gonzalez et al., 2019). This study highlighted a closely related behavior of ISCs in both 3D *in vitro* and *in vivo* platforms based on the expression profile of the genes leucine-rich repeat-containing G protein-coupled receptor 5 (Lgr5), SOX9 Lrig HOPX. It also allowed to establish that ISCs are likely to play a critical role during the reparative events that occur after IRI. In addition, it is suggested that the ISCs-mediated epithelial repair mechanism, probably depends on the degree of epithelial loss produced by the injury.

In a different approach, spheroid culture was used to provide exosomes to ameliorate kidney IRI damage in C57BL/6 male mice (Zhang, X. et al., 2021). Using a 3D platform allowed the provision of a higher number of exosomes, with defined size, compared with 2D. The exosomes produced, were harvested from culture media and then administrated *via* renal intra-capsular, within a 72 h reoxygenation period. Histological, immunohistochemical, and ELISA analyses of kidney samples were performed to assess cell death and inflammation; serum creatinine and urea nitrogen were measured to assess kidney function. The study concluded that the strategy managed to significantly attenuate renal injury in AKI mice, alleviate tissue apoptosis, and decrease pro-inflammatory cytokine production.

In general, spheroid-based models are fit for single-cell, short-term cultures, molecular evaluation, and drug screening. Nonetheless, when long-term culture and/or multi-cellular approaches are required, their limitations become evident. Spheroid growth is concomitant with the formation of heterogeneous environments, uncontrolled oxygen/nutrient gradients, and thus inconsistent proliferation slopes, and even necrotic areas (Zhang, X. et al., 2021). The critical thickness for viable spheroid culture has been established by several authors to be around 150–200 μm and up to 25 days long (Katt et al., 2016; Shi et al., 2018). To tackle these limitations, scaffold-based models become necessary.

## Scaffold-Based Models and IRI

**Figure 5:**
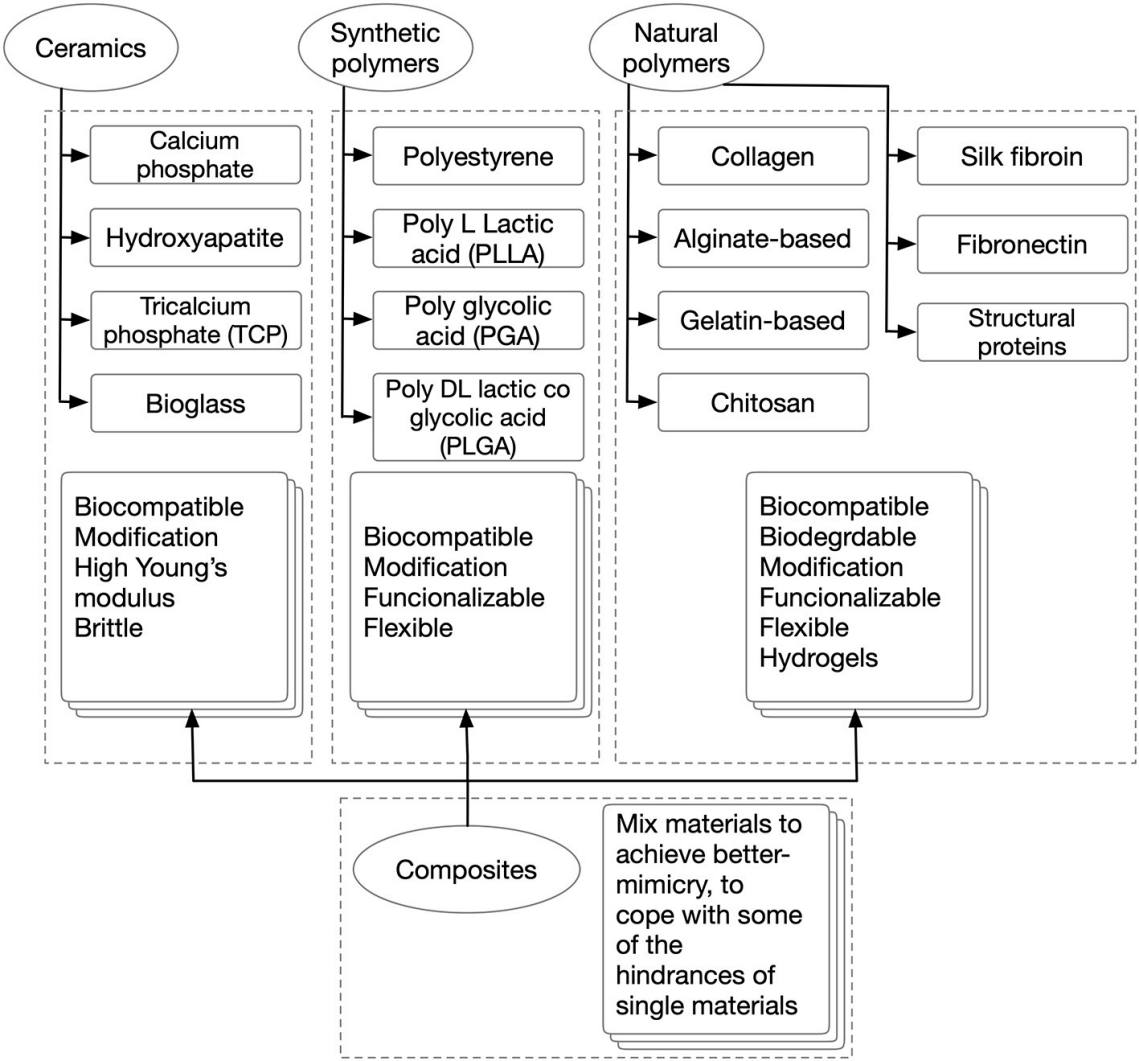
Scaffold biomaterials.

In scaffold-based 3D cultures, cells are grown, and developed anchored to biocompatible 3D structures that resemble the ECM of the tissues they are devised to mimic. These matrices are designed to supply cells wherein an auxiliary and often temporary niches were to adhere, migrate, grow, and differentiate for ECM deposition. Consequently, scaffolds define a template for normal cell-cell interactions, cell-ECM interactions, and specify the mass transfer coefficient for nutrients and signal delivery. Thus, their functionalization to drive cell fate, attract cells from the surroundings, and form a direct interface between the scaffold and the injury site is desired (Zanoni et al., 2016). Scaffolds are restrictive semi-permeable environments, as cells get physically isolated from the exterior, which allow the diffusion of essential molecules and leave outside the undesired ones (Nicolas et al., 2020). Also, these provide shape, internal structure, pore architecture, mechanical properties, and a place for neovascularization and neo-tissue formation (Uludag et al., 2000). In other words, scaffolds play the role of an artificial 3D ECM. Interestingly, the aforementioned features share a degree of customizability through the selection of materials and fabrication strategy. Scaffolds can be temporary, biocompatible, and bioresorbable matrices that are slowly resorbed by cells during their growth and development, either *ex* or *in vivo* or permanent, synthetic cell niches. Their selection is strongly defined as per the tissue to mimic and the application. There is a wide range of materials with desirable features that have been studied, such as acellularized-ECM, ceramics, and polymers from natural and synthetic origins. See Figure 5 for a comparison between different scaffold biomaterials. Different manufacturing and post-processing approaches allow the modulating of parameters such as cell-binding sites, surface topography, thickness, porosity, pore size, shape, tortuosity, interconnectivity, mechanical properties, and scaffold shape (Uludag et al., 2000; Chan and Leong, 2008). Electrospinning, air-jet spinning, centrifugal spinning, salt leaching, freeze-drying, stereolithography, self-assembly, phase separation, gas foaming, melt molding, fiber mesh, solvent casting, photo-polymerization, laser sintering, encapsulation, and 3D bioprinting are some of the scaffold fabrication techniques available. For a more in-depth investigation of scaffold fabrication techniques, the readership should refer to several excellent reviews (Angelopoulos et al., 2020; Haider et al., 2020). This tunability is key for tailor-making specific scaffolds features for each ischemia/IRI tissue to model. This is a very important aspect of this platform; unique aspects of different tissues/organs can be provided. Thus, these come in different forms, from simple-shaped organoids, and hydrogels, to more complex architectures like 3D bioprinted. In addition, scaffold-based 3D constructs have the advantage to allow the generation of single-cell or multi-cellular models. Consequently, these allow better-mimicry of the cellular heterogeneity found *in vivo* in regular tissues (Loh and Choong, 2013).

## Organoids

An organoid is a self-organized 3D cellular platform in which a simple miniature version of tissues is provided. As such, they are engineered to display specific micro-anatomical features from the tissues they mimic. Organoids are the closest scaffold-based models to the spheroid platform. However, their use allows the establishment of long-term cultures. In a study made to recapitulate the intestinal epithelium, six cycling Lgr5^+^ SCs obtained from the small intestinal crypt from mice, produced 3D cultured in Matrigel® organoids (Gupta et al., 2020). It was possible to reach a 1.5 years-long culture, that formed basic mini-guts with crypt-villus physiology without a mesenchymal niche. Wingless-related integration site (Wnt), EGF, and Noggin modulation were critical for this feat. Organoids can be fabricated by techniques like suspension culture, magnetic cell levitation, ECM hydrogel/scaffold-based, i.e., Matrigel®, Culturex BME, alginate, etc., and spinning-flask culture. Herein, cells are attached, which then develop into mini tissues. Depending on cell type, these could form embryoid bodies prior to their final development (Sato et al., 2009). Such platforms have been used to study the mechanism of IRI, the role of SCs in the regeneration process, and the production of biologics with potential therapies for the treatment of renal (Ashok et al., 2020; Shiva et al., 2020), hepatic (Kishi et al., 2019), and intestinal (Chen et al., 2016; Matsumoto et al., 2017; Zhou et al., 2017; Stieler Stewart et al., 2018; Koike et al., 2020; Kip et al., 2021), cardiovascular, lung, skin flap, fat, and neural (Chen et al., 2014) reperfusion damage.

One related study featured proteomic profiling of human intestinal organoids models during hypoxia and reoxygenation, to study the protein dynamics and molecular mechanisms of IRI (Kip et al., 2021). Interestingly, the model featured a differentiated organoid phenotype provided with two defined regions, crypt, and villus, and presence of mucus-containing goblet cells and enterocytes. This was confirmed by Alcian blue and Alizarin red staining, which was further confirmed by the upregulation of I-FABP and MUC2 and a deregulation of OLF4 compared with undifferentiated, among other such techniques. The model allowed the study of the differential molecular response of these two defined regions. The organoid was selectively induced to produce either a villus-like or a crypt-like phenotype. After reoxygenation, the most significantly enriched biological process in both phenotypes was related to the energy production in the mitochondria and the mitochondrion organization process. Several subunits of the mitochondrial respiratory chain complexes were altered. Protein translation was enhanced in the crypt-like group (increment of multiple 60 S ribosomal proteins of RPL4, RPL6, RPL7a, RPL13, RPL35, RPL18, etc.), while it was reduced for villus-like (RPL13, RPL4, etc.). Protein synthesis and ribosome biosynthesis are energy-consuming routes that get repressed during hypoxia to save energy. With the reoxygenation onset, these processes restart differentially for both systems due to different proliferative stages of the villus- and crypt-like organoids. Processes associated with lipid metabolism were enriched for both models. For instance, several proteins involved in fatty acid β-oxidation showed a decreased expression, a mechanism related to cell death. In addition, increased transcript levels of the HIF1A target gene, VEGF confirmed hypoxic signalling, and support regulation of cellular stress signalling for both villus- and crypt-like. In addition, a relationship between the hypoxic stress was established, as regulation of immune response-related processes was observed despite the lack of immune cells in the system. Thus, this study validates the models developed as adequate platforms to study IRI response, as their *in vitro* behavior was in concordance with what is seen *in vivo*.

Another study introduced the use of an iPSCs-produced neural organoid model to assess the therapeutic potential of exosomes produced from neural progenitor cells (NPCs) from newborn mice following experimental strokes *in vitro*, and to compare the effect *in vivo* (Chen et al., 2014). Their results highlighted how exosomes significantly reduced cell injury in organoids when compared with controls, in dosage-dependent effect. These were then applied *in vivo*, where enhanced neurological recovery and neuroregeneration were seen for as long as 3 months. Analyses of blood and brain samples 7 days post-ischemia displayed an increase in the concentration of lymphocytes B and T in blood, without affecting cerebral cell counts.

These studies support the idea that patient-based organoids can capture cellular heterogeneity, as well as the cellular niche from tissues, recapitulating the cellular phenotypes from the donor. Thus, these provide an adequate mimicry to post-stroke immunosuppression and represent a decent contender to close the gap between the *in vitro* and *in vivo* podiums. However, organoids are not free from constraints because their shape, size, internal structures, and interactions are not true to tissues. Thus, there are several aspects of tissue homeostasis and *in vivo* principles that are not fulfilled properly. Organoid cultures yield cell constructs with unsatisfactory cell density, fate, and heterogeneity (especially compared to adult tissue). These further yield unmatched mass transport properties, lack of vascularization, and interorgan communication (Zheng et al., 2021). To cope with these limitations, creating a more sizable template which able to capture the complexities of tissues is required.

## Sizeable Scaffolds and IRI

Polymeric soft scaffolds, foams, and hydrogels can be built through most of the techniques described above. Throughout these methods, tissue constructs can be designed in more complex shapes, compared with spheroids and organoids (Kim et al., 2020). In addition, composite scaffolds and multicellular tissues can be manufactured. Such platforms have been used to study the mechanism of IRI, the role of SCs in the regeneration process, and to produce biologics with potential therapies in renal (Dankers et al., 2015; Soranno et al., 2016; Liu et al., 2020), hepatic (Gao et al., 2012; Xiao et al., 2018), cardiovascular (Ahmad et al., 2013; Song et al., 2014; Shan et al., 2017; Xiao et al., 2017; Li et al., 2021; Xing et al., 2021; Zhang, Y. et al., 2021), lung, skin (Salimath et al., 2012), retinal (Uehara et al., 2019), and neural (Lee et al., 2012) reperfusion damage. Still, the versatility of techniques used to manufacture and functionalize scaffolds pushes the boundaries found in organoid and spheroid methods, and allows for the development of wider applications.

Injectable hydrogels containing biotherapeutic agents were used to pioneer treatment for chronic myocardial infractions *in vivo* in rat models (Xiao et al., 2017). Hyaluronic acid hydrogels loaded with Stromal-derived factor-1 (SDF-1) and N-acetyl-seryl-aspartyl-lysyl-proline (Ac-SDKP) were injected directly into the ischemic area. The proteins used in the scaffold were selected to provide stem cell homing an angiogenic effect, ultimately aiming to regenerate the infarction and lessen IRI. After 4 weeks of treatment, histology and immunostaining showcased a significant increase in myocardial regeneration (including the increased formation of arterioles), cell recruitment, and recovery of heart function, while preventing heart hypertrophy, compared with no treatment or the application of single factors (SDF-1 or SDKP). However, the mechanisms that produced such results were not established and more research should follow. Another related study developed a polycaprolactone-based fibrous scaffold *via* electrospinning which is capable of releasing functional biomolecules in a controlled manner under physiological conditions (Rajkovic et al., 2018). The device was designed to deliver hydrogen sulfide (a signaling molecule with cytoprotective action) directly into the injury site to protect cells from ROS damage. These studies highlight the relevance of scaffold design, and functionalization, to improve the *in vivo* healing factor of scaffold-based implants.

A composite scaffold consisting of decellularized human myocardium, with preserved microarchitecture and biomechanics, and fibrin hydrogels, seeded with TGF-β-conditioned human MSCs, was established. The study was conducted *in vitro*, and in a nude rat *in vivo* model. Molecular, structural, and functional parameters were assessed (Feng et al., 2015). The scaffold was devised considering large, interconnected pores and smooth channels to promote a prompt permeation of cells. In addition, TGF-β-conditioning of cells was used to aid the formation of neovasculature throughout the pore network to encourage an overall better integration to the surrounding tissues. Their *in vivo* results displayed cell migration into the infarct bed and the scaffold; recovery of the ability of cells to secrete SDF-1. Consequently, cell migration was further improved, as well as revascularization of the infarct bed and recovery of tissue function.

A tuneable size multicellular scaffold consisting of decellularized human lung fibroblast-derived matrix (hFDM) and collagen hydrogel and containing human umbilical vein endothelial cells (HUVECs) and MSCs, was established *in vitro*. The model was engineered with the aim to evaluate its therapeutic efficacy for IRI treatment in a mouse hindlimb ischemia model (Godier-Furnémont et al., 2011). In this platform, multiple angiogenic-related factors were significantly upregulated, compared to single-cell scaffolds. In addition, the treatment significantly improved blood reperfusion and reduced the fibrosis level of the ischemic tissue. These studies highlight the importance of adding complexity to our models through composite materials and a multicellular approach to producing implant-grade tissue mimetics.

An interesting strategy to accomplish an *in vitro* ischemic culture onset would be to couple a rapid oxygen consumption reaction to the scaffold fabrication stage. For instance, the study of Lewis et al. (2017) devised a protocol that links a laccase-mediated crosslinking reaction to the production of a gelatin-based hydrogel that is suitable to mimic such ischemic start (Du et al., 2020). Thus, an oxygen gradient is established when reperfusion begins; such an oxygen gradient depends on the distance from oxygen-carrying blood vessels.

In a different approach, ischemic preconditioning (IPC), a non-invasive drug-free procedure normally used to ameliorate the ischemic insult, and IRI has been used to promote vascularization and increase the TE therapy success. Ischemic preconditioning consists of providing transitory periods of ischemia to tissues, to prepare them for the subsequent insult (Lewis et al., 2017). In this process, endogenous repair mechanisms are induced by the action of several paracrine, angiogenic factors, such as VEGF, and nitric oxide synthase. Additionally, IPC has been seen to provide an SCs homing effect (Tomai et al., 1999; Lim et al., 2012; Wang et al., 2018). The study of Lim et al. (2012) engineered polyacrylic chambers positioned surrounding the femoral vessels of adult Sprague-Dawley rats, where fibrin scaffolds seeded with neonatal rat ventricular cardiomyocytes (rCMs) were implanted. The chambers were able to provide a controlled IPC setting, thus allowing to study the effect of IPC in the development of TE neo-tissues *in vivo*. Their results displayed that IPC applied to empty chambers allowed the femoral vessels to naturally deposition a fibrin scaffold. Inflammatory cells, fibroblasts, pericytes, and vascular progenitors migrated heterogeneously to the scaffold; they were assembled into vascularized granulation tissues in due time. When IPC was applied to chambers with cell-seeded scaffolds, the developed tissue had higher weight and volume, which then produced higher neo-vascularization and cardiac muscle volume. In addition, rCMs were protected from apoptosis (Tomai et al., 1999). These results, along with other similar studies (Bo et al., 2013; Chen and Vunjak-Novakovic, 2019), sustain the idea that IPC or *in vitro* hypoxia preconditioning could be key for increasing the success of TE therapies.

Rigid scaffolds and IRI have not been studied significantly to include them here. However, this does not imply that such platforms could not be useful in the field. Rigid porous scaffolds are commonly studied in TE, as these provide adequate mimicry for bone implants. Furthermore, models for bone diseases research and bone IRI models/implants could be established with such materials. Between these materials, graphene and bio-inspired ceramics, e.g., bioactive glass, β-tricalcium phosphate, hydroxyapatite, calcium silicate, *etc*., are commonly studied (Oh et al., 2010).

Although traditional scaffold manufacturing techniques have been successful in providing numerous solutions and tissue-mimicry, the difficulty to scale fully functional scaffold-based organs for critical-size therapy is still in demand. In order to disentangle the true potential of scaffold-based models, it is fundamental to address the challenges that arise. These include adequate inner architecture (controlled pore size, porosity, tortuosity, and pre-vasculatures), mass transport properties, full-thickness, external shape-accuracy, and mechanical properties (Gao et al., 2014).

## 3D Bioprinted Scaffolds and IRI

Undoubtedly, the search for the perfect tissue-mimicry would require a technique able to solve the mentioned challenges; 3D bioprinting seems to be the adequate technology for the task. Bioprinting is the assembly of 3D arrangements with cell-laden biomaterials in defined architectures in a layer-by-layer approach. It is a versatile additive manufacturing-based tool which allows the production of heterogeneous scaffolds with high resolution and control, that is based on computer-aided design models (CAD) and on patient imaging. It allows the manufacture of constructs that assume the external shape of tissues and organs, with their internal structure and pore microarchitecture (Chan and Leong, 2008). In addition, bioprinting allows certain regulation over the transport properties of scaffolds throughout the selection of materials and curing methods. Moreover, by tailoring multi-material composite geometries with defined pores (defined pore quantity, tortuosity, and size), microchannels and/or regions with different densities and multiple tissue-specific features can be achieved (Chan and Leong, 2008). In addition, different deposition methods provide versatility, including inkjet bioprinting, extrusion bioprinting, laser-assisted bioprinting, multiphoton excitation based fabrication, spheroid extrusion, spheroid bio-assembly, and 4D bioprinting, the use of stimuli-responsive materials (Mohanty et al., 2016).

Since the creation of this technique ~15 years ago, it has been used as a platform to generate functional tissue models to study regeneration, for drug screening, and as a disease model. Nonetheless, it has yet to be established as a platform to study IRI. Numerous research groups have been working to establish bioprinted platforms, especially in regenerative medicine and cancer research, to create clinically accurate *ex vivo* tissues and tumor models of different tissues. Their efforts have produced studies related to neovascularization, hypoxia, as well as oxygen-releasing scaffolds (Farina et al., 2017; Ong et al., 2018; Erdem et al., 2020; Sun et al., 2020), that support the idea that bioprinting could improve IRI models tissue-mimicry, especially to minimize preclinical trials.

## Microfluidic Systems/Bioreactors/Organ-on-A-Chip and IRI

**Figure 6:**
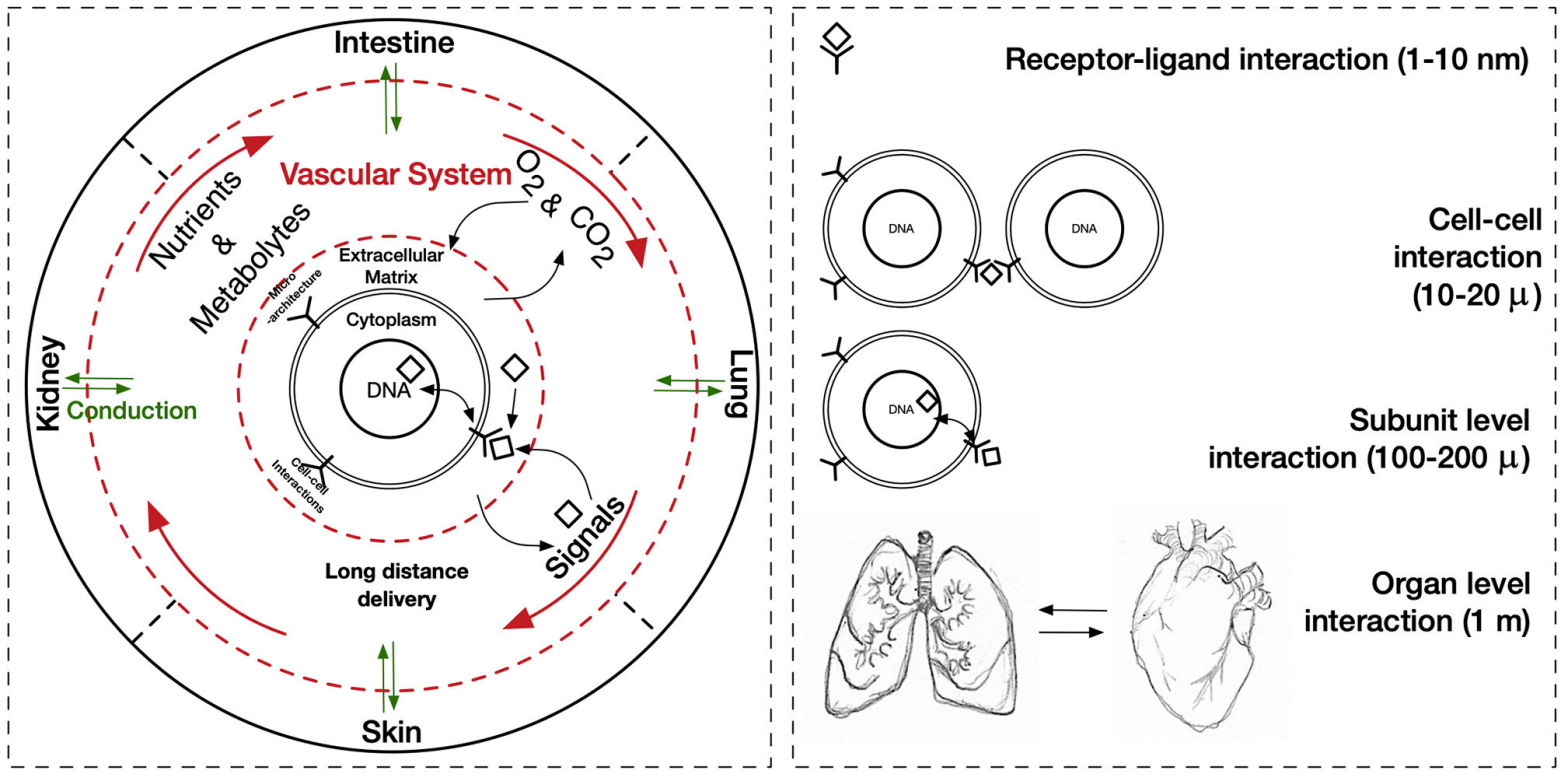
Global interactions involved in tissue homeostasis and its scale.

In order to unravel the entire potential of the TE approach and its full-fledged contribution to support IRI studies, it is fundamental to cater to yet another extra layer of mimicry. Hitherto, it has been established as a platform that considers the cellular material involved in IRI, the microarchitecture for these cells to inhabit and the interactions these cells would have in this synthetic residence. The last frontier in mimicry would be placing these neo-tissues in a system that depicts the normal macroscopic or global Interorgan communication, as well as the blood flow. This, then, provides a holistic model that recapitulates all aspects of physiological IRI, in patient-derived models, with the capability to provide the homeostasis of healthy tissue, and the hypoxia and reoxygenation involved in IRI. In Figure 6, schematics portraying the global interactions of tissues are shown. This level of interactions could be implemented with media perfusion and the delivery of controlled culture conditions and biophysical cues. Media perfusion could be accomplished by employing microfluidic devices; while the delivery of controlled conditions and signals by the use of bioreactors. This induces cues through scaffolds that mimic interorgan interactions, blood flow, blood flow-induced shear stress, and providing yet another source of local transport properties tunning, i.e., oxygen and nutrients. Additionally, this setting allows waste removal of the culture (Giraud et al., 2020). Perfusion could be performed through an open or a closed loop system, as well as throughout gas permeable tubes. Oxygenators and/or gas-permeable membranes could be provided for better oxygenation. Flow mode could be modulated to be steady, pulsatile, or oscillated. These, then produce dynamic shear stress, hydrostatic pressure, mechanical modulation of SCs, and tailored diffusivity (Adamo and García-Cardeña, 2011; Estrada et al., 2011; Ali et al., 2020). In addition, biophysical cues, such as optic and magnetic/electric, could be provided to culture chambers to further influence SCs fate (Wang et al., 2020). This consequently minimize the use of expensive biochemical signals. One related model was established by Chen and Vunjak-Novakovic; it was used for the study of human myocardial IRI (Bo et al., 2013). The model utilized hydrogel-encapsulated hIPS-differentiated cardiomyocytes cultured in a tissue engineering bioreactor. Their study managed to mimic IRI, showing through cell death, histology, immunostaining, and western blot how ischemia damaged the established normal tissue and how reperfusion further increased the damage. In this setting, different cardioprotective strategies were evaluated. A similar study which used bioreactor-based IRI models was able to recapitulate important hallmarks of IRI, considering cardiomyocyte viability, disruption of cellular ultrastructure, angiogenic potential and secretion of key proangiogenic, and pro-inflammatory cytokines. Furthermore, quantitative whole-proteome analysis was performed to assess the injury, upregulation of proteins associated with migration, proliferation, paracrine signaling, and stress response-related pathways; these were observed when compared with the control condition (Sebastião et al., 2020).

Establishing a platform like the ones described above, especially when sizeable or bioprinted scaffolds are used, could require large quantities of biological materials, more so cues could be very time-consuming and expensive. Thus, its application in patient-specific studies could be challenging; rendering this platform is mostly useful for long-term research projects. Organ-on-a-chip technologies, or multi-channeled 3D microfluidic cell culture chips, can be provided with all the aforementioned in a micro-sized setting. Thus, these are able to deliver patient-specific whole-organ functionality devices in a fast-paced, more reproducible, and cheaper pipeline, compared with bioreactors. This platform would provide an adequate platform for the assessment of the effect of target drugs or treatments for IRI. Such a strategy has been used to establish a porcine cardiomyocytes-based heart-chip able to assess the effect of shear stress in IRI (Khanal et al., 2011). The setting was able to provide the tissue with an ischemic state, to then deliver reperfusion in a controlled manner. The study followed mitochondrial membrane potential, early-stage apoptosis, cell adhesion, and morphology of cells. A cycle of 4 h-long ischemia, followed by a 2 h-period of reperfusion, produced the cell blebbing formation typically seen in physiological IRI.

In another study, a microfluidic IRI model was established utilizing a vascular compartment containing human endothelial cells, which allowed its obstruction with human blood clots and then its re-perfusion *via* thrombolytic treatment (Nemcovsky Amar et al., 2019). This novel IRI model provided an ischemia and reperfusion mechanism closely related to what is seen *in vivo*. Thus, it represents a key feature to include in future embolic based IRI models. Such design assets could allow in the future the better understanding of IRI and the potential service as platforms for the development of novel therapeutic methodologies.

In general, multi-channeled chips can be established to assess several treatments in a single experiment. As such, these represent a very interesting scenery for the study of patient-specific IRI and a simplified version of the full-size bioreactor pipeline. Both settings represent the staple technologies that TE could deliver to serve the purpose of IRI research.

See Table 1 for a summary of the presented strategies, and the technological approaches to enhance their success.

**Table 1:** Summary of none-scaffold- and scaffold-based strategies and the technological approaches to enhance their success.

**None-scaffold-based**		
Exploit the cell-to-cell adhesion features of SCs. Cells form 3D constructs which lack any type of anchorage medium and are mainly comprised of ECM.		
**Spheroids**	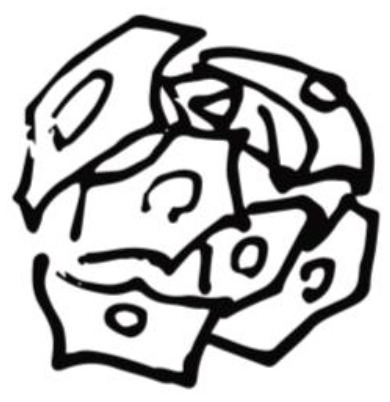	
Micro-size cell aggregates. None-anchorage medium. Spheroid size affects oxygen availability.		
**Pros:**	Cons:	
Adequate for single-cell, short-term cultures, For molecular evaluation, and drug screening. Scalability, reproducibility, and minimize manual-handling.	Not adequate for long-term culture, and/or for multi-cellular approaches.	
	Formation of heterogeneous environments, uncontrolled oxygen/nutrient gradients, inconsistent proliferation slopes, and necrotic areas.	
Critical thickness for viable spheroid culture has been established around 150–200 um, and up to 25 days-long.		
**Scaffold-based**		
Cells are grown and developed anchored to 3D ECM-like constructs.		
Template for normal cell-cell interactions, cell-ECM interactions.		
Specific mass transfer coefficient for nutrients, and signal delivery		
**Organoids**	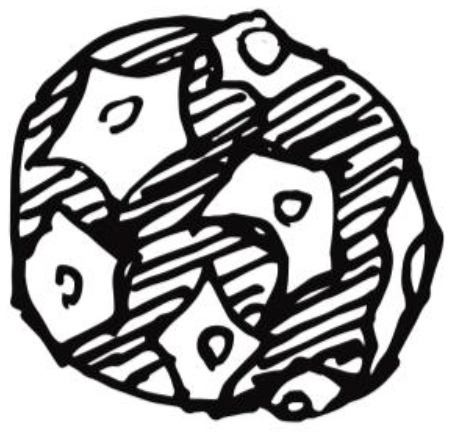	
Self-organized 3D scaffold. Simpler, miniature version of a tissue. Closest models to the spheroid.		
**Pros:**	Cons:	
Multicellular, long-term cultures. Formation of specific micro-anatomical features. Scalability, reproducibility, and minimize manual-handling.	Shape, size, internal structure, and interactions are not true to tissues.	
	tissue-homeostasis and ***in vivo*** principles are not fulfilled properly.	
	Cell-constructs with unsatisfactory cell density, fate, and heterogeneity.	
	Unmatched mass transport properties.	
	Lack of vascularization.	
	Lack of interorgan communication.	
Critical thickness for viable organoid culture has been established around 400–450 um, and up to 1.5 years-long, forming basic mini-tissue-features (e.g., crypt-villus physiology)		
**Sizable scaffolds**	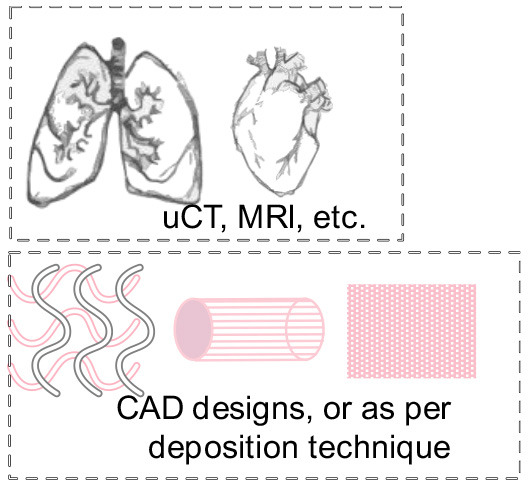	
Provide shape, internal structure, pore architecture, mechanical properties, and a place for neovascularization. Functionalisation.		
Pros:	Cons:	
Multicellular, long-term cultures. Formation of anatomical features. Soft, rigid, or composite materials. Allows to provide controlled heterogeneity	Less scalability, reproducibility.	
	More manual-handling.	
	Cells grow slower due to impeded transport properties and interactions	
Critical thickness and duration of culture are defined specifically by the macrostructure of the construct and application, formation of multiple tissue-features, and potentially full-organs.		
**Strategies that improve mimicry**		
Complex sizable scaffolds require technology		
**3D Bioprinting**	**Bioreactors**	**Microfluidics**
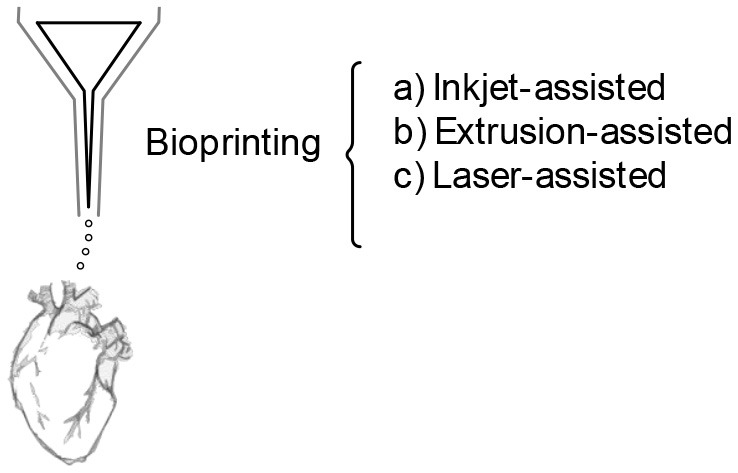	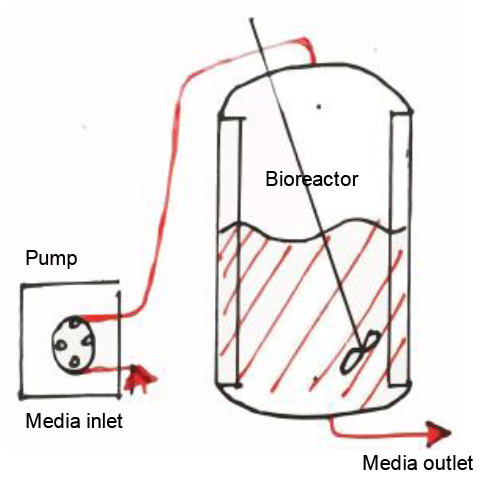	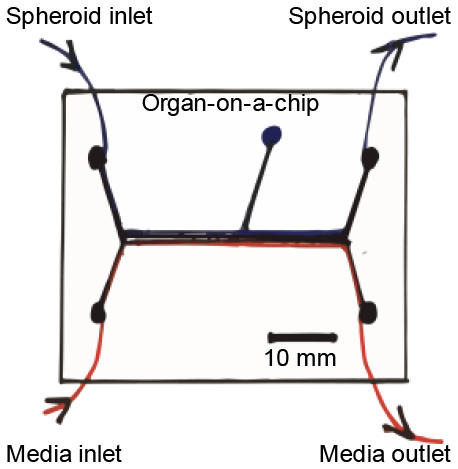
Assembly of cell-laden multi-material heterogenous scaffolds Defined architectures in a layer-by-layer approach. High resolution, and control CAD models/ patient imaging. External shape, with internal structure.	Systems that emulate the macroscopic/global Interorgan communication, as well as the blood flow.	
	Holistic models that recapitulate all aspects of physiological IRI	
	Hypoxia, and reoxygenation involved in IRI	

## Final Remarks and Conclusions

Comparisons between the 2D and 3D platforms have systematically given credit to the postulate that 3D environments provide a better-matched mimicry to the *in vivo* tissues. The same can be said for dynamic culture over static culture. This is a novel platform to tackle with *in vitro* IRI protocols. As such, there is plenty of space for future research and many aspects of this condition have to be addressed. In particular, to address the consideration of the unique aspects of IRI associated with different organs, as well as multiorgan responses. Tissue engineering has made sufficient research to provide tissue-mimics that could be adjusted to research IRI and its organ- and tissue-specific aspects. Selection of cells, scaffold materials, and differentiation cues represent a start. In addition, different types of research call for providing platforms with akin levels of complexity. Spheroids could be used to perform fast screening studies for patients in a clinical setting, while organoids could bring an extra layer of mimicry for longer studies that require cellular heterogeneity. More intricacy could be provided through the design of internally- and externally-accurate composite scaffolds, allowing the highest level of mimicry while providing a template for angiogenesis. In addition, an *ex vivo* ischemia and reperfusion onset that resembles the *in vivo* homologous condition has to be established through hypoxia and normoxia culture cycles. A strategy combining bioprinting, culture chambers, and microfluidic systems would allow the delivery of closer-to-physiology devices and increase the sophistication of the fundamental research that the *in vitro* setting could provide. This strategy would provide both tailor-made and off-the-shelf organs that could be stored in cell banks to be ready-available for treatments, and research on demand (Sato and Clevers, 2013). Through this review, different settings were able to address different research outcomes, as well as different IRI platforms for different tissues have been discussed. Thus, establishing the great level of customizability that follows the TE bid on IRI. However, the flexibility it presents, along with the heterogeneity of all the organs that can suffer IRI, is concomitant with innumerable design choices, cell, and biomaterial selection, type of scaffold, biological, and physical cues, fluid flow profiles, etc. Henceforth, thorough testing to ensure reproducibility, quality, and safety of the products has to be done, and specific configurations for each tissue/organ have to be established. Medical devices must adhere to stringent regulatory frameworks and quality standards of production to translate into future clinical therapeutics/therapies.

## Author Contributions

MZ wrote this review study. All authors revised and approved the manuscript.

## Funding

This study was supported by Santander Universia, Universidad de Chile, and FAPESP UFRO 2020/06982-3.

## Conflict of Interest

The authors declare that the research was conducted in the absence of any commercial or financial relationships that could be construed as a potential conflict of interest.

## Publisher's Note

All claims expressed in this article are solely those of the authors and do not necessarily represent those of their affiliated organizations, or those of the publisher, the editors and the reviewers. Any product that may be evaluated in this article, or claim that may be made by its manufacturer, is not guaranteed or endorsed by the publisher.

## Glossary

ATP: Adenosine triphosphate
ROS: Reactive oxygen species
NRS: Reactive nitrogen species
IRI: Ischemia-reperfusion injury
NO: Nitric oxide
TE: Tissue engineering
MAPK: Mitogen-activated protein kinase
NFκB: Nuclear factor kappa-light-chain-enhancer of activated B cells
ERK1/2: Extracellular signal-regulated kinase ½
JNKs: c-Jun N-terminal kinases
HIF: Hypoxia inducible factor
PAS: Per-ARNT-Sim
ODD: Oxygen-dependent degradation domain
PHD-2: Proline-hydroxylase-2
TAD: Transactivation domains
VEGF: Vascular endothelial growth factor
PKC: Protein kinase C
NADPH: Nicotinamide adenine dinucleotide phosphate
NOX: NADPH oxidase
AMPK: AMP-activated protein kinase
NOS: Nitric oxide synthase
Dusp: Dual-specificity phosphatase
Adm: Adriamycin
IL-1b: Interleukin 1 beta
EGF: Epidermal growth factor
Hbegf: Heparin-binding EGF-like growth factor
Socs3: Suppressor of cytokine signaling 3
Cos2: Costal2
NFκbiz: NFκB inhibitor, zeta
Btg2: BTG Anti-Proliferation Factor 2
Rhob: Rho-related GTP-binding protein
Tfpi2: Tissue factor pathway Inhibitor 2
Verge: Vascular early response gene
Plk2: Serine/threonine polo like kinase 2
Kif6: Kinesin family member 6
Nr4a1: Nuclear receptor subfamily 4 group A member 1
Jun: Transcriptional factor Jun
JunB: Transcriptional factor JunB
Fos: Transcriptional factor Fos
Atf3: Activating Transcription Factor 3
ERK5: Extracellular signal-regulated kinase 5
p38: Protein kinase p38
JNK: Jun n-terminal kinase.
